# Supplementary material for: Gate-tunable plasmons in mixed-dimensional van der Waals heterostructures
Source: Nat Commun. 2021 Aug 19;12:5039. doi: 10.1038/s41467-021-25269-0 (PMC8376888; doi:10.1038/s41467-021-25269-0)
Supplement: Supplementary file 1 — Supplementary Information for Gate-tunable plasmons in mixed-dimensional van der Waals heterostructures [file 41467_2021_25269_MOESM1_ESM.pdf]

**Supplementary Information**

# Gate-tunable plasmons in mixed-dimensional van der Waals heterostructures

Sheng Wang[1,2†*], SeokJae Yoo[1,3†*], Sihan Zhao[1†], Wenyu Zhao[1], Salman Kahn[1], Dingzhou Cui[4], Fanqi Wu[4], Lili Jiang[1], M. Iqbal Bakti Utama[1,2,5], Hongyuan Li[1,6], Shaowei Li[1,2], Alexander Zibrov[1,2], Emma Regan[1,2,6], Danqing Wang[1,2,6], Zuocheng Zhang[1], Kenji Watanabe[7], Takashi Taniguchi[8], Chongwu Zhou[4,9], Feng Wang[1,2,10*]

[1]Department of Physics, University of California at Berkeley, Berkeley, California 94720, USA.

[2]Materials Sciences Division, Lawrence Berkeley National Laboratory, Berkeley, California 94720, USA.

[3]Department of Physics, Korea University, Seoul 02841, Korea.

[4]Department of Chemical Engineering and Materials Science, University of Southern California, Los Angeles, California 90089, USA.

[5]Department of Materials Science and Engineering, University of California at Berkeley, Berkeley, California 94720, USA.

[6]Graduate Group in Applied Science and Technology, University of California at Berkeley, Berkeley, California 94720, USA.

[7]Research Center for Functional Materials, National Institute for Materials Science, 1-1 Namiki, Tsukuba, 305-0044, Japan.

[8]International Center for Materials Nanoarchitectonics, National Institute for Materials Science, 1-1 Namiki, Tsukuba, 305-0044, Japan.

[9]Department of Electrical Engineering, University of Southern California, Los Angeles, California 90089, USA.

[10]Kavli Energy NanoScience Institute at the University of California, Berkeley and the Lawrence Berkeley National Laboratory, Berkeley, California, 94720, USA.

[†]These authors contributed equally to this work.

*To whom correspondence should be addressed.

Email: shengwang16@berkeley.edu; seokjaeyoo@berkeley.edu; fengwang76@berkeley.edu

## Supplementary Note 1: Optical conductivity of graphene

We use the well-established optical conductivity formula of graphene for the theoretical predictions and the numerical simulations in the main text. The optical conductivity of graphene at an excitation frequency $\omega$ and a Fermi energy $E_F$ has two contributions[1,2],

$$\sigma\left(\omega,E_F\right)=\sigma_{\text{intra}}\left(\omega,E_F\right)+\sigma_{\text{inter}}\left(\omega,E_F\right), \quad \text{(S1)}$$

where the intraband term is given by

$$\sigma_{\text{intra}}\left(\omega,E_F\right)=\frac{2e^2}{\pi\omega}\frac{k_BT}{\hbar}\ln\left[2\cosh\left(\frac{E_F}{2k_BT}\right)\right]\frac{i}{\omega+i/\tau}, \quad \text{(S2)}$$

and the interband electron transition term is given by

$$\sigma_{\text{inter}}\left(\omega,E_F\right)=\frac{e^2}{4\hbar}\left[H\left(\frac{\omega}{2}\right)+i\frac{4\omega}{\pi}\int_0^\infty d\varepsilon\frac{H(\varepsilon)-H(\omega/2)}{\omega^2-4\varepsilon^2}\right], \quad \text{(S3)}$$

with the Boltzmann constant $k_B$, the temperature $T$, the reduced Planck constant $\hbar$, and the relaxation time $\tau$. The function $H(\omega)$ is defined by

$$H(\varepsilon)=\frac{\sinh\left(\hbar\varepsilon/k_BT\right)}{\cosh\left(\mu/k_BT\right)+\cosh\left(\hbar\varepsilon/k_BT\right)}. \quad \text{(S4)}$$

In this work, we use $T = 300$ K and $\tau = 100$ fs. The relaxation time $\tau = 100$ fs is estimated from the experimental plasmon study of the bare graphene region in the heterostructure.

## Supplementary Note 2: Diameters of SWNT M1 and M2 determined from AFM height profiles

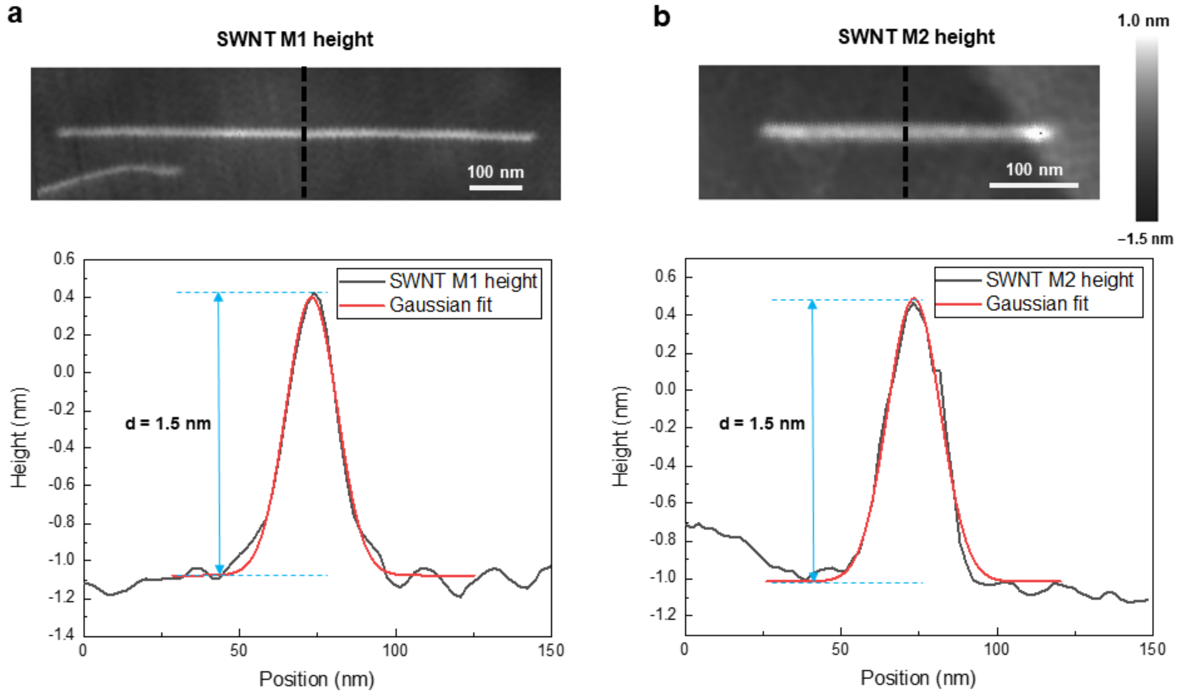


**Supplementary Figure 1| Diameters of SWNT M1 and M2 determined from AFM height profiles.** **a**, Topography of SWNT M1 obtained simultaneously with the near-field images. Height profile along the black dotted line across SWNT M1 is presented as the black curve in the lower panel. The red curve is Gaussian fitting to the height profile, from which the diameter of SWNT M1 is determined to be $d$ = 1.5 nm. **b**, Topography of SWNT M2 obtained simultaneously with the near-field images. Height profile along the black dotted line across SWNT M2 is presented as the black curve in the lower panel. The red curve is Gaussian fitting to the height profile, from which the diameter of SWNT M2 is determined to be $d$ = 1.5 nm.

## Supplementary Note 3: Infrared nano-imaging of graphene plasmons and analysis

Infrared nano-imaging is performed at the region near the graphene edge to visualize graphene plasmons. The gate-dependent plasmon interference patterns near the graphene edge are shown in Supplementary Fig. 2. In the low-doping regime (Supplementary Figs. 2d to 2f), graphene plasmons suffer from severe interband transition loss evidenced by the low visibility of interference fringes near the edge. At higher gate voltages on both the electron side (Supplementary Figs. 2b, 2c) and the hole side (Supplementary Figs. 2g to 2k), prominent fringes emerge near the graphene edge, which correspond to well-defined plasmonic excitations. The near-field profiles are averaged normal to the graphene edge in the white dashed area outlined in Supplementary Fig. 2b. The profiles and analysis at various gate voltages are summarized in Supplementary Fig. 3. We can extract the plasmon wavelength $\lambda_p$ and quality factor $Q$ by fitting the profiles with a damped oscillator form $e^{-2\pi x/(Q\cdot\lambda_P)}\sin\left((4\pi x)/\lambda_p\right)$. The dependence of plasmon wavelength on gate voltage is depicted by the red dots in Supplementary Fig. 3d. The relaxation time $\tau$ is estimated to be 100 fs from the extracted quality factor. We can also obtain the corresponding fast Fourier transform (FFT) amplitude spectra (Supplementary Fig. 3b) from the real-space profiles (Supplementary Fig. 3a). The peaks of the FFT amplitude spectra correspond to the half plasmon wavelength, $\lambda_p/2$. The plasmon wavelengths $\lambda_p$ obtained by FFT is represented by the black squares in Supplementary Fig. 3d, which is consistent with the results from fitting (red dots in Supplementary Fig. 3d).

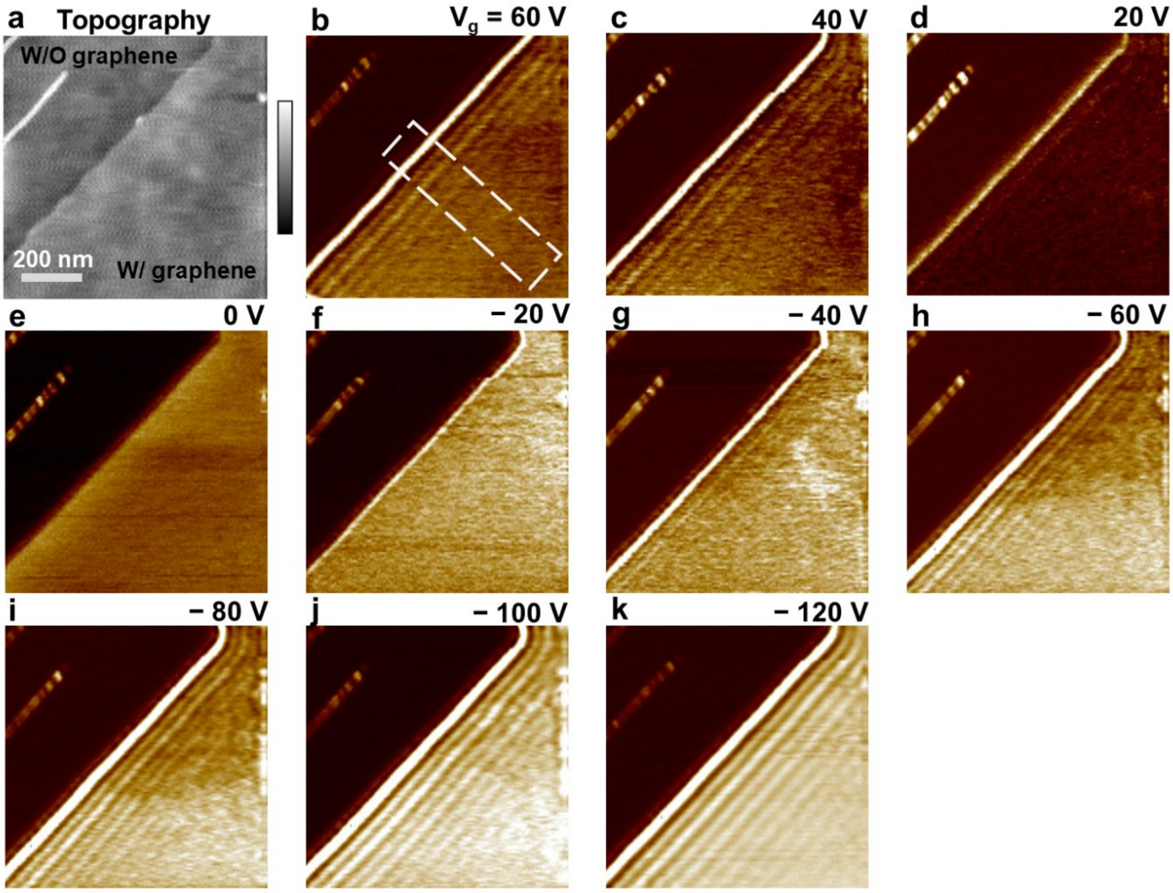

**Supplementary Figure 2| Infrared nano-imaging of graphene plasmons near the edge of graphene in the SWNT/h-BN/graphene heterostructure. a**, Topography of the region near graphene edge. The regions with and without graphene are noted. **b** to **k**, Gate-dependent plasmon interference patterns at various gate voltages. The near-field profiles are averaged normal to the graphene edge in the white dashed area outlined in **b**. The profiles and analysis at various gate voltages are summarized in Supplementary Fig. 3.

In theoretical and numerical calculations, the optical responses of graphene are calculated in terms of the graphene Fermi energy $E_F$ according to eq. (S1). The Fermi energy $E_F$ is associated with the experimental back-gate voltage $V_g$ by the capacitance model $E_F = \hbar v_F \sqrt{\pi C_g |V_g - V_{cnp}|}$, where $\hbar$ is the reduced Planck's constant, $v_F$ is the Fermi velocity, $C_g$ is the back-gate capacitance density, $V_{cnp}$ is the charge neutral point. $V_{cnp}$ is estimated to be $-5$ V from the infrared nano-imaging results shown in Supplementary Fig. 2. The back-gate capacitance density $C_g$ is estimated to be $6.7 \times 10^{10}$ $e\text{cm}^{-2}\text{V}^{-1}$ ($e$ is the elementary charge) for the heterostructure with a $SiO_2$ layer of 285 nm and a bottom h-BN layer of 40 nm between graphene and the conductive Si layer. Graphene plasmon wavelength $\lambda_p$ depends on graphene Fermi energy $E_F$ as $\lambda_p = \left(e^2 |E_F|\right) / \left(\varepsilon_0 \varepsilon_{med} \hbar^2 \omega^2\right)$, where $\varepsilon_0$ is the vacuum permittivity, $\varepsilon_{med}$ is the relative permittivity of the surrounding medium[3]. We can determine the corresponding Fermi energy $E_F$ for different gate voltages $V_g$ from the gate-dependent plasmon wavelengths $\lambda_p$ experimentally obtained at each $V_g$. The extracted $V_g$ - $E_F$ dependence is displayed by the black squares in Supplementary Fig. 3c. Gate voltage $V_g$ is related to $E_F$ as $V_g = V_{cnp} + \frac{1}{\hbar^2 V_F^2 \pi C_g} E_F^2$. Therefore, we fit the obtained $V_g$ in terms of $E_F$ using the formula $V_g = a + bE_F^2$, with fitting parameters $a = V_{cnp}$, $b = \frac{1}{\hbar^2 V_F^2 \pi C_g}$. Fitted curve for the $V_g$ - $E_F$ relation is plotted by a red solid line in Supplementary Fig. 3c. From the fitting parameter b, we can obtain an effective capacitance density to be $6.6 \times 10^{10}$ $e\text{cm}^{-2}\text{V}^{-1}$. This matches well with the capacitance density estimated from the geometric capacitor model. The theoretical graphene plasmon wavelength in terms of the converted gate voltage $V_g$ agrees well with the experimental values as shown in Supplementary Fig. 2d. We use the $V_g$ - $E_F$ relation in Supplementary Fig. 3c in the main text to make a direct comparison between theory and experiment.

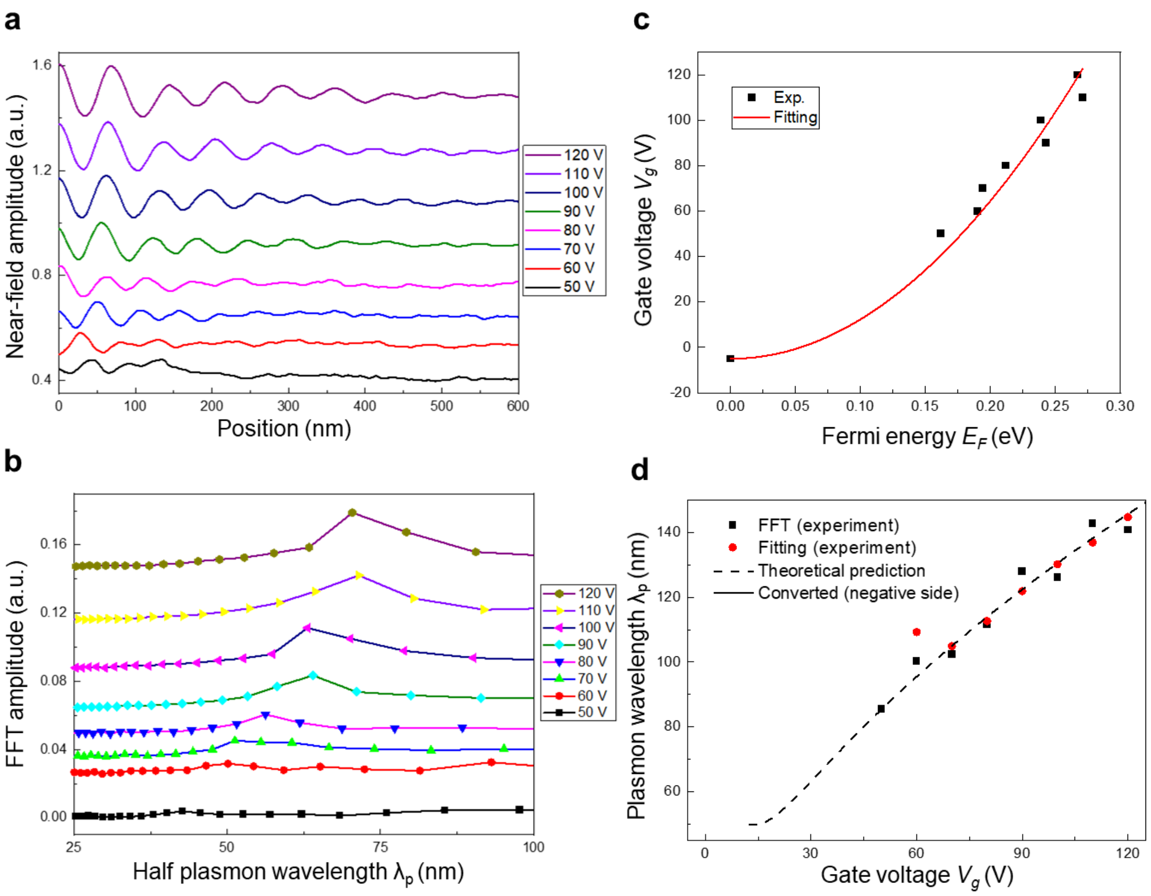


**Supplementary Figure 3| Graphene plasmon analysis. a**, Near-field amplitudes averaged normal to the graphene edge in the white dashed area outlined in Supplementary Fig. 2b at various gate voltages. **b**, Corresponding fast Fourier transform (FFT) amplitude spectra in the momentum space from the real-space profiles in **a**. **c**, Gate voltage $V_g$ as a function of Fermi energy $E_F$ extracted from the plasmon wavelength. **d**, Comparison of theoretically predicted graphene plasmon wavelength (dashed line) with the experimentally obtained plasmon wavelengths (red dots: fitting results, black squares: FFT results). Note that a.u. in **a** and **b** denotes arbitrary units.

## Supplementary Note 4: Derivation of the Fresnel-like graphene response function *r*(*k*, *E*F)

We start from the Fresnel reflection coefficient for the plane waves to derive the graphene response function to the evanescent waves. The 2D conducting sheet with the optical conductivity σ(*ω*, $E_F$) is sandwiched by a semi-infinite (1) dielectric medium and (2) an anisotropic medium. The anisotropic medium has a permittivity tensor in the form,

$$\boldsymbol{\varepsilon}_2 = \varepsilon_0 \begin{pmatrix} \varepsilon_{2,\parallel} & 0 & 0 \\ 0 & \varepsilon_{2,\parallel} & 0 \\ 0 & 0 & \varepsilon_{2,\perp} \end{pmatrix}. \tag{S5}$$

This is consistent with our experimental device which has a thick h-BN bottom layer. For p-polarization with the xz-plane of incidence, the reflection coefficient for the plane wave is given by[4]

$$r = \frac{\varepsilon_1 k_{2z,e} - \varepsilon_{2,\parallel} k_{1z} + k_{1z} k_{2z,e} \eta_0 \sigma / k_0}{\varepsilon_1 k_{2z,e} + \varepsilon_{2,\parallel} k_{1z} - k_{1z} k_{2z,e} \eta_0 \sigma / k_0}. \tag{S6}$$

with the vacuum impedance $\eta_0 = \sqrt{\frac{\mu_0}{\epsilon_0}}$, where $\mu_0$ and $\epsilon_0$ are vacuum permeability and permittivity, respectively. Note that the normal direction to the medium interface is z-direction. The normal momentum of e-ray in the anisotropic medium 2 satisfies the relation,

$$k_{2z}^2 = \varepsilon_{2,\parallel} k_0^2 - \frac{\varepsilon_{2,\parallel}}{\varepsilon_{2,\perp}} \beta^2, \tag{S7}$$

with $\beta = k_1 \sin\theta$, $k_{1z} = k_1 \cos\theta = \sqrt{\varepsilon_1} k_0 \cos\theta$, $k_0 = \omega / c$, and the incident angle $\theta$.

To convert the Fresnel reflection coefficient, eq. (S6), into the response function to the evanescent wave, we straightforwardly replace the normal components of the wave momenta, $k_z \to -ik_z$ and $k_{ex} = -ik_{ez}$. It yields

$$r(\omega, E_F) = -\frac{\varepsilon_{2,\parallel} k_{1z} - \varepsilon_1 k_{2z.e} + ik_{1z} k_{2z,e} \eta_0 \sigma(\omega, E_F) / k_0}{\varepsilon_{2,\parallel} k_{1z} + \varepsilon_1 k_{2z,e} + ik_{1z} k_{2z,e} \eta_0 \sigma(\omega, E_F) / k_0}. \tag{S8}$$

The transverse momenta $\beta$ becomes the plasmon momentum $k_p$ as well. The normal momentum in the medium 2 correspondingly has the form,

$$k_{2z}^2 = \frac{\varepsilon_{2,\parallel}}{\varepsilon_{2,\perp}} k_p^2 - \varepsilon_{2,\parallel} k_0^2. \tag{S9}$$

Importantly, the pole of the graphene response function, eq. (S8), gives the graphene plasmon dispersion relation on anisotropic substrates,

$$\frac{\varepsilon_{2,\parallel}}{k_{2z,e}}+\frac{\varepsilon_1}{k_{1z}}=-\frac{i\eta_0}{k_0}\sigma\left(\omega,E_{\mathrm{F}}\right). \tag{S10}$$

Solving eq. (S10) for the complex-valued $k_{\mathrm{p}}$, we obtain the bare graphene plasmon wavelength and quality factor in Fig. 3. Note that eq. (S11) gives the approximated form for the graphene plasmon momentum in the non-retarded regime,

$$k_{\mathrm{p}}\approx\frac{1}{2}\left(\varepsilon_1+\sqrt{\varepsilon_{2,\parallel}\varepsilon_{2,\perp}}\right)\sqrt{\frac{\varepsilon_{2,\parallel}}{\varepsilon_{2,\perp}}}\frac{2ik_0}{\eta_0\sigma}. \tag{S11}$$

## Supplementary Note 5: Derivation of the hybrid plasmon dispersion

We solve the potential problem of a cylindrical wire on a substrate with 2D conducting graphene sheet at the interface. For the wire, we use cylindrical coordinate $(\rho,\theta,z)$ whose z-axis corresponds to the wire axis. The axial components of the Hertz vector potential for the wire are given by[5]

$$\begin{aligned}\psi_{in}(\rho,z) &= A_0 e^{ik_p z} I_0\left(\sqrt{k_p^2-k_{in}^2}\rho\right)\\ \psi_{wire}(\rho,z) &= B_0 e^{ik_p z} K_0\left(\sqrt{k_p^2-k_{med}^2}\rho\right)\end{aligned}, \tag{S12}$$

with the modified Bessel function $I_n$ and $K_n$ of the order $n$. $A_0$ and $B_0$ are the coefficients of potential which are determined by the external source such as an electric dipole source mimicking the tip or a plane wave. We only consider the lowest order $n = 0$ because of a thin wire. In presence of the substrate and the 2D conducting sheet, we need to include the potential of the reflected light. To obtain the analytic form of the potential of the reflected light, we can expand the modified Bessel functions in $\psi_{wire}(\rho,z)$ in terms of the plane and evanescent waves with a spectrum of the wave momenta $k$. This technique is called the angular spectrum of the modified Bessel function[6,7]. It enables us to write

$$KW_m(\xi,\eta) \equiv K_m(k\rho)e^{im\theta} = \int_{-\infty}^{+\infty} \frac{\left(q+\sqrt{1+q^2}\right)^m}{2\sqrt{1+q^2}} \exp\left(-\xi\sqrt{1+q^2}\right)\exp(i\eta q)\,dq, \tag{S13}$$

where the new coordinates are given by $\xi = k\rho\cos\theta$ and $\eta = k\rho\sin\theta$. If the wire is suspended on the interface at a height $h$, the potential of the reflected light ($RW$) in the region $\xi < 2\chi$ can be written in terms of the response function $r$.

$$RW_m(\xi,\eta) = \int_{-\infty}^{+\infty} r\left(\sqrt{1+q^2}\xi, q\eta\right)\frac{\left(q+\sqrt{1+q^2}\right)^m}{2\sqrt{1+q^2}} \exp\left\{-(2\chi-\xi)\sqrt{1+q^2}\right\}\exp(i\eta q)\,dq, \tag{S14}$$

with $\chi = k_\rho h$. Note that $r$ is the ordinary Fresnel reflection coefficient for the plane wave components ($q < k_0$), but it becomes the Fresnel-like response function to the evanescent wave components ($q \geq k_0$).

To further simplify the potential of the reflected light, we adopt the following approximations, namely a resonant momentum approximation:

1) The dominant contribution in the integration originates from the point $q = 0$ and other contributions from nonzero q decays exponentially due to the term $\exp\left(-\xi\sqrt{1+q^2}\right)$.
2) Graphene plasmons are resonantly excited by the wire potential $\psi_{wire}(\rho,z)$ with the momentum $k_p$.

3) The boundary condition at the wire surface $\rho = R$ is only applied to the surface facing to graphene, that is $\theta = 0$. Therefore, we can use $\eta = 0$.

Adoption of these approximations allows us to write

$$RW_0(\xi,\eta) = \frac{1}{2} r(\xi) e^{-(2\chi-\xi)}. \quad \text{(S15)}$$

Therefore, we can approximate the potential of the reflected light as,

$$\psi_{ref}(\rho,z) = \frac{1}{2} r\left(\sqrt{k_p^2 - k_{med}^2}\right) B_0 e^{ik_p z} e^{-\sqrt{k_p^2-k_{med}^2}(2h-\rho)}. \quad \text{(S16)}$$

The tangential electric field $E_z$ and magnetic field $H_\theta$ are related to the potential $\psi$ as $E_z = \left(k_p^2 - k^2\right)\psi$ and $H_\theta = \left(ik^2/\mu\omega\right)\left(\partial\psi/\partial\rho\right)$. At the wire surface, $\rho = R$, we can apply the boundary conditions to resolve the dispersion relation for the hybrid plasmon momentum $k_p$, which is shown as eq. (1) in the main text. We also note that that the plasmon dispersion relation (eq. 1) in the limit $r(k) = 0$ reduces to that of bare SWNTs[8]. We are also able to write the plasmon dispersion explicitly in the following form, which is equivalent to eq. 1 in the main text when we use the approximation:

$$\frac{\varepsilon_{in}}{\varepsilon_{med}} \frac{k_{\rho,med}}{k_{\rho,in}} = -\frac{I_0\left(k_{\rho,in}R\right)}{I_1\left(k_{\rho,in}R\right)} \frac{K_1\left(k_{\rho,med}R\right) - RW\left(k_p, E_F\right)}{K_0\left(k_{\rho,med}R\right) + RW\left(k_p, E_F\right)}. \quad \text{(S17)}$$

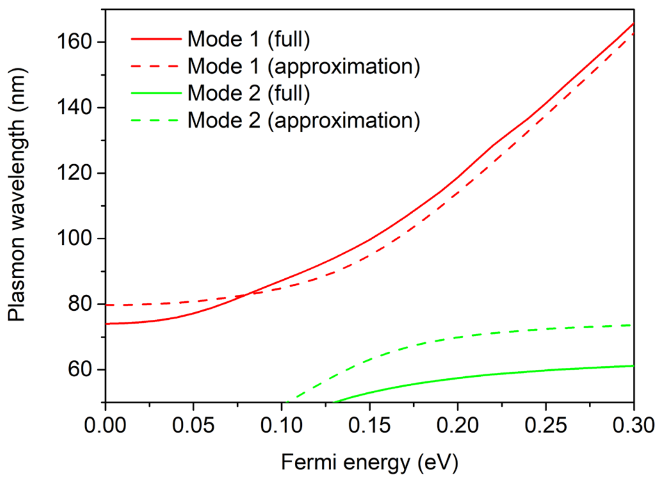


**Supplementary Figure 4| Comparison between the approximated and the full-wave expressions of the reflected Bessel function.** Plasmon wavelengths of mode 1 (red) and mode 2 (green) are calculated by the dispersion using the full-wave (solid) and the approximated (dashed) expressions for the reflected Bessel function $RW_0$, respectively.

In Supplementary Fig. 4, we calculate the hybrid plasmon modes using the plasmon dispersions (eq. (S17)) from the approximated (eq. (S15)) and the full-wave (eq. (S14)) expressions. We use the SWNT permittivity as $\varepsilon_{in}/\varepsilon_{med}$ = -210 + 23.52$i$ and -300 + 16.8$i$ for the approximated results (dashed lines) and the full calculated results (solid lines), respectively.

This permittivity is chosen to yield the similar plasmon wavelength at $E_F = 0$ eV. Note that numerical results in Fig. 4 of the main text used $\varepsilon_{in}/\varepsilon_{med} = -300 + 16.8i$ which is the same as the full calculation. As shown in Supplementary Fig. 4, the plasmon wavelength of the upper mode 1 remains the same, but that of the lower mode 2 becomes slightly shorter.

To investigate the difference between the approximated and the full-wave expressions, we decompose the region of $k_p$, that is solution of the plasmon dispersion (eq. (S17)), into two categories: resonant and off-resonant regions of graphene response function $r(k,E_F)$ according to different $E_F$ values. Eq. (S14) is composed of the Gaussian-like lineshape function, $\exp\left\{-(2h-R)\sqrt{1+q^2}k_{\rho,med}\right\}/\left(2\sqrt{1+q^2}\right)$ centered at q = 0 and the graphene reflection coefficient for the evanescent waves, $r(k,E_F)$. When the hybrid plasmons are graphene-like (the mode 1 above $E_F > 0.13$ eV), the graphene response function $r$ is also resonant at $q = 0$, which justifies the $q = 0$ approximation. When the hybrid plasmon momentum $k_p$ is far from graphene plasmon momentum (the mode 1 below $E_F < 0.13$ eV and the mode 2), the graphene reflection coefficient $r\left(\sqrt{1+q^2}k_{\rho,med}\right)$ is off-resonant in the $q$-space. To see the effect of the off-resonant $r\left(\sqrt{1+q^2}k_{\rho,med}\right)$, we can expand eq. (S14) in the leading order of $(k_pR)$ as follows:

$$\varepsilon_{in}/\varepsilon_{med} = -\frac{1}{K_0\left(k_pR\right)+RW\left(k_p,E_F\right)}\frac{2}{\left(k_pR\right)^2}. \qquad \text{(S18)}$$

In the off-resonant region, $RW_0\left(k_{\rho,med}R\right)$ has the Gaussian-like lineshape with negative values for all $q$'s. Taking the approximation $RW_0\left(k_{\rho,med}R\right) \approx r\left(k_p\right)e^{-(2h-R)k_p}/2$ yields smaller negative values, resulting in smaller $\varepsilon_{in}/\varepsilon_{med}$ in eq. (S18) than the full calculation including all $q$'s.

## Supplementary Note 6: Numerically simulated plasmon modes for different SWNT diameter *d* and gap size *h*

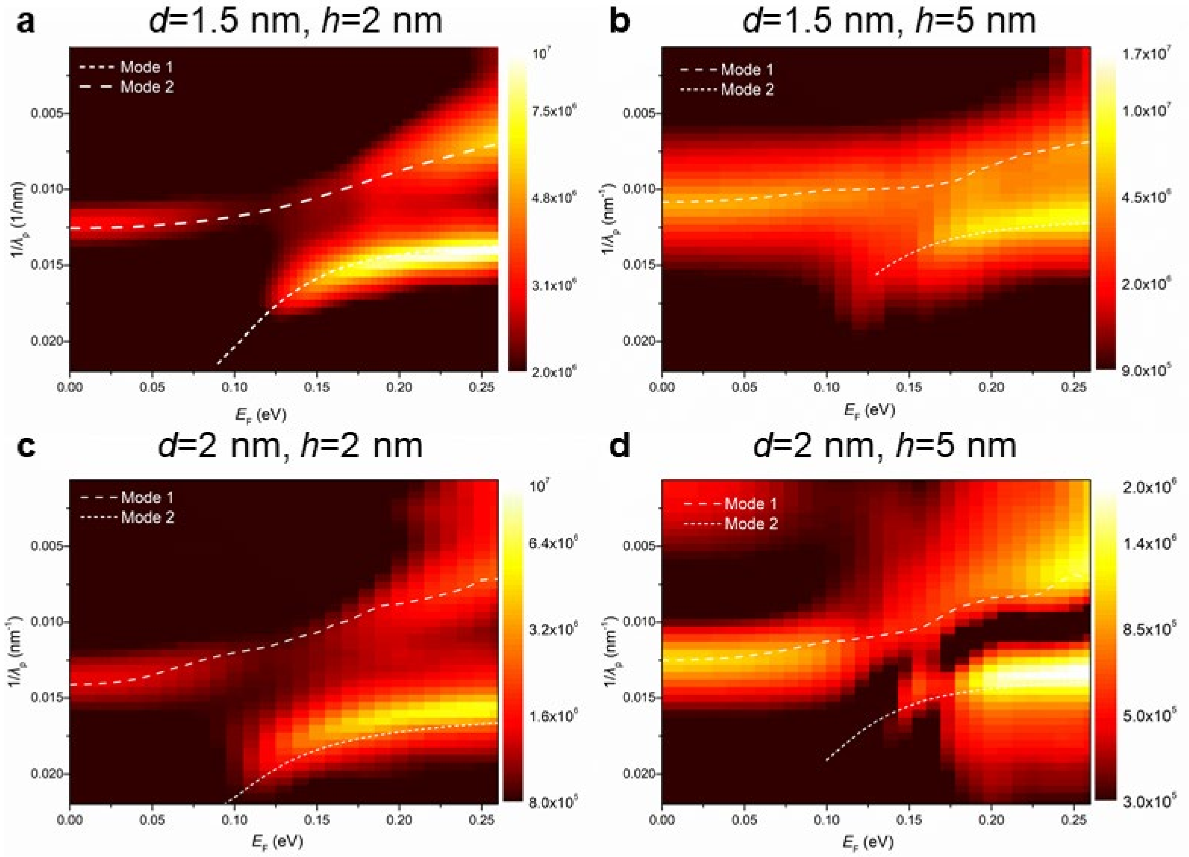


**Supplementary Figure 5| Numerically simulated plasmon modes for different SWNT diameter *d* and gap size *h*.** Fourier transform amplitudes of the radial electric field, $|\mathrm{Re}(E_z)|$ at the bottom surface of SWNT for different SWNT diameter *d* and gap size *h*: **a**, $d = 1.5$ nm, $h = 2$ nm, **b**, $d = 1.5$ nm, $h = 5$ nm, **c**, $d = 2$ nm, $h = 2$ nm, and **d**, $d = 2$ nm, $h = 5$ nm. White dashed lines in **a** to **d** are the analytical prediction from Eq. (S17). Note that **a** is the same data as Fig. 4a in the main text.

We perform theoretical analysis and numerical simulations on the SWNT/h-BN/graphene heterostructure to investigate the effects of the SWNT diameter *d* and the gap size *h* between SWNT and graphene on plasmon hybridization. We calculated four cases: (a) $d = 1.5$ nm, $h = 2$ nm, (b) $d = 1.5$ nm, $h = 5$ nm, (c) $d = 2$ nm, $h = 2$ nm, and (d) $d = 2$ nm, $h = 5$ nm. As shown in Supplementary Fig. 5, different diameters *d* and gap size *h* can have sizable effects on the plasmon hybridization, including the crossing point and the coupling strength. The coupling strength is encoded in the plasmon mode splitting amplitude after hybridization. Case (c) with parameters $d = 2$ nm, $h = 2$ nm (Case (b) with parameters $d = 1.5$ nm, $h = 5$ nm) features the largest (smallest) mode separation, indicating the largest (smallest) coupling strength among the four studied cases. With larger SWNT diameter *d*, the radial electric field distribution of SWNT plasmon becomes more spatially extended. The mode overlap between SWNT and graphene plasmons increases with decreasing gap size. Therefore, larger SWNT diameter and smaller gap size lead to stronger mode coupling. This is also evident in our analytic model. Eqs. (S14) to (S16) show that the coupling strength largely exhibits the exponential scaling with the round-trip distance between the SWNT surface and graphene as $\sim\exp[-k_p(2h-R)]$. The

coupling strength for the four cases thus rank as (c)>(a)>(d)>(b), which is consistent with the mode splitting amplitudes by the theoretical and numerical results. When the gap size *h* approaches the quantum tunneling threshold ($h$ < 0.5 nm), the dependence of coupling strength on gap size can show deviation from the above analysis.

The crossing point can also be affected by the SWNT diameter *d* and the gap size *h*. As the SWNT diameter *d* becomes larger, the bare SWNT plasmon wavelength $\lambda_p$ becomes slightly smaller[9]. As the gap size between SWNT and graphene becomes smaller, the screening effects of graphene on SWNT becomes larger. Larger SWNT diameter and smaller gap size thus results in the decrease of the SWNT plasmon wavelength at $E_F$ = 0 eV. At $E_F$ = 0 eV, $\lambda_p$ is 70.8 nm for case (c) in Supplementary Fig. 5c, but $\lambda_p$ becomes 92.3 nm for case (b) in Supplementary Fig. 5b. The increase (decrease) in SWNT plasmon wavelength at $E_F$ = 0 eV eventually shifts the crossing point of SWNT and graphene plasmons to (higher) lower $E_F$. The crossing point $E_F$ of the four cases rank as (c) < (a) < (d) < (b), exhibiting a reverse order compared with the coupling strength.

We note that the full-wave numerical results in Supplementary Fig. 5 also validate our analytic results in the Supplementary Information section 5 because the analytic results (white dashed lines) match well with the numerical results (colored profiles). For the case of $d$ = 1.5 nm, $h$ = 2 nm in Supplementary Fig. 4 and Supplementary Fig. 5a, it is also seen that the approximated analytic results obtained by Eq. (1) in the main text shows reasonable agreement with the full-wave numerical results.

We would like to remark that random phase approximation method within the Fermi liquid theory will predict the same plasmon dispersion behaviors in metallic SWNTs and the same hybrid modes in the van der Waals heterostructures as those obtained by the Luttinger liquid theory[10,11]. Therefore, the measurements of the plasmons in metallic SWNTs and the hybrid plasmons in the van der Waals heterostructures cannot provide a definitive proof of the Luttinger liquid by itself. To unambiguously demonstrate the Luttinger liquid behavior, one can combine the study of nanotube plasmons determining the Luttinger liquid parameter *g* with the electrical tunneling study showing a power-law scaling in the tunneling conductance with an index determined by *g*[12]. This correlation of plasmon study and electrical tunneling measurements, together with other theoretical and experimental work in the literatures, can provide a complete understanding of the Luttinger liquid and the mixed dimensional system[12-14].

## Supplementary Note 7: Measurability of the hybrid plasmon modes

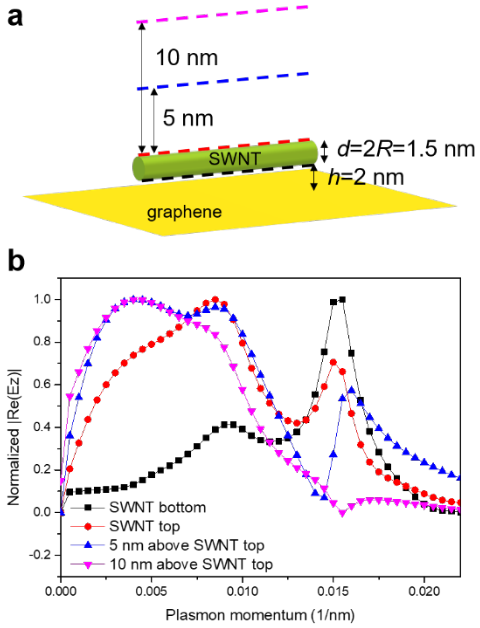


**Supplementary Figure 6| Different electric field distribution of the two hybrid plasmon modes. a**, Schematic illustration of four measurement heights (black, red, blue, and magenta dashed lines). **b**, Spectra of the normalized FFT amplitudes of Re($E_z$) corresponding to the field measurement positions in **a** at $E_F$ = 0.17 eV.

Both analytic theory (Fig. 3 in the main text) and numerical simulation (Fig. 4a in the main text) predict the existence of two modes due to the plasmon hybridization. The lower mode branch is not clearly resolved in our experimental infrared nano-imaging results. The absence of clear lower plasmon mode in the experimental data is likely to arise from a combination of the following two effects.

First, for the lower plasmon mode, the out-of-phase charge oscillations in SWNT and graphene leads to a shorter plasmon wavelength and the local concentration of the electric field between SWNT and graphene rather than on top of SWNT, resulting in weaker coupling of this mode to the metallic AFM tip. Supplementary Fig. 6 shows the numerically obtained normalized FFT spectra of Re(Ez) at four different measurement heights. We can find that the relative strength of the lower mode (that is the mode with shorter plasmon wavelength) becomes weaker when we measure Re(Ez) at higher positions. At the gap (the SWNT bottom with black line in Supplementary Fig. 6), the lower mode peak is stronger than the upper mode peak, indicating strong gap localization of the lower plasmon mode. Our SNOM experimentally measures the near field at an average height of ~ 40 nm on top of SWNT, and therefore the contribution from the lower mode becomes vanishingly small compared to upper mode. We note that the absence of the lower plasmon mode in the experimental infrared nano-imaging results has also been previously reported in a h-BN separated graphene bilayer structure[15].

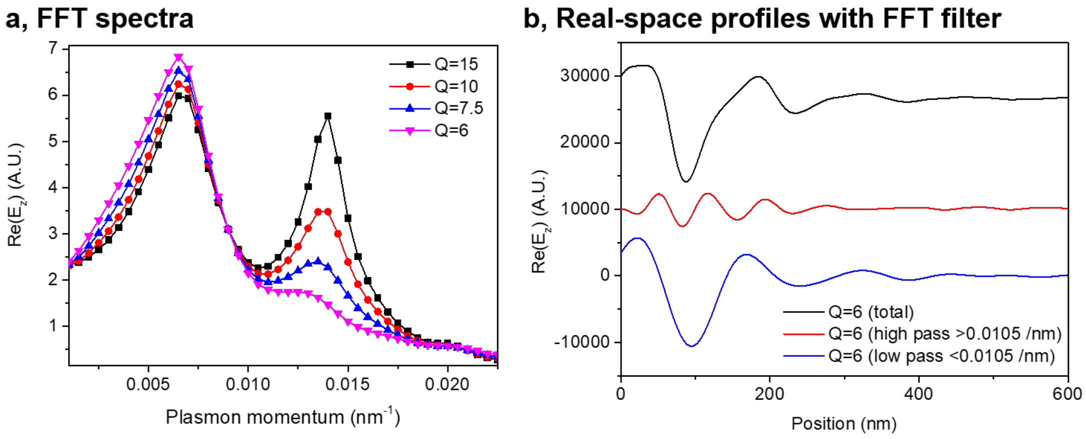


**Supplementary Figure 7| Comparison between the two hybrid plasmon modes. a,** FFT spectra of Re($E_z$) on the SWNT surface at $E_F$ = 0.26 eV with different Q factors for SWNT plasmons. **b,** Real-space profiles for Re($E_z$) when SWNT plasmons have Q = 6. The plasmons are excited by a z-directed dipole source at the end of SWNT and 150 nm above the SWNT top. Low and high pass FFT filters for the total Re($E_z$) (black line) enable the extraction of mode 1 (blue line) and 2 (red line), respectively. Note that A.U. in **a** and **b** denotes arbitrary units.

Second, the quality factor of the lower plasmon branch is smaller than the higher branch. As shown in the simulated results in Supplementary Fig. 7, the upper plasmon mode 1 can largely dominate the plasmon response when the SWNT plasmon quality factor is moderate. Numerical simulations with different Q factors are shown in Supplementary Fig. 7. The ratio between the imaginary and real part of the SWNT permittivity are set to be -Im($\varepsilon_{in}$)/Re($\varepsilon_{in}$) = 0.112, 0.168, 0.224, and 0.280 for Q = 15, 10, 7.5, and 6, respectively. Note that Q = 15 corresponds to Fig. 4 in the main text. The real part of the SWNT permittivity is fixed to be Re($\varepsilon_{in}$) = -210. As the SWNT Q factor decreases, in FFT spectra (Supplementary Fig. 7a), mode 1 (upper branch) becomes slightly stronger while mode 2 (lower branch) becomes weaker. At an exemplary quality factor Q = 6, Supplementary Fig. 7b shows that the real-space profile of the radial fields Re($E_z$) is dominated by the mode 1 rather than the mode 2, which can lead to the unobserved lower mode in the real-space nano-imaging results.

## Supplementary Note 8: Gate-tunable plasmons in SWNT M1 and M2

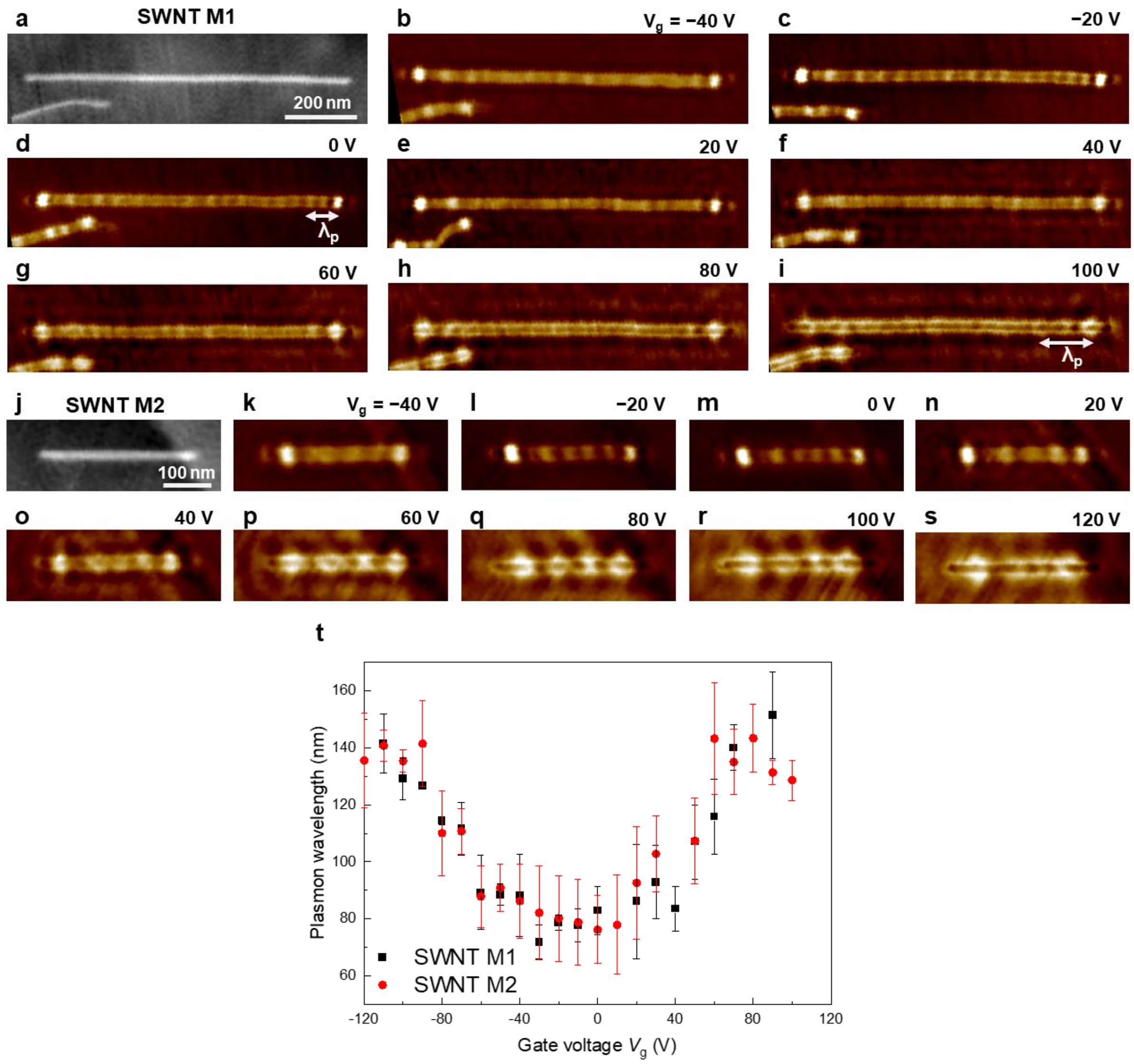


**Supplementary Figure 8| Gate-tunable plasmons in SWNT M1 and M2. a**, Topography of SWNT M1. **b** to **i**, Corresponding near-field images of SWNT M1 at various gate voltages from −40 to 100 V. We clearly observe that prominent near-field oscillations emerge near the right nanotube end, and that they depend sensitively on the gate voltage. The plasmon wavelength $\lambda_p$, equal to twice the oscillation period in the near-field images, is marked by the white double arrow (**d** and **i**) and becomes longer as the gate voltage is varied further away from 0 V. **j**, Topography of SWNT M2. **k** to **s**, Corresponding near-field images of SWNT M2 at various gate voltages from −40 to 120 V. SWNT M2 acts as a Fabry-Perot plasmonic nanocavity where propagating plasmons are reflected back and forth by both ends and produce a collective response. From **m** to **s**, the number of antinodes decreases from 7 to 4. **t**, The algorithmically extracted plasmon wavelengths versus the gate voltage $V_g$ at both positive and negative gate voltages (black dots: SWNT M1, red dots: SWNT M2). The error bars represent the standard deviation of the averaged peak-to-peak distances in plasmon oscillations along SWNTs. At the positive side of $V_g$, we observe largely symmetric tunable plasmon wavelengths compared with those at the negative side of $V_g$.

**Supplementary References:**


1. Falkovsky, L. & Pershoguba, S. Optical far-infrared properties of a graphene monolayer and multilayer. *Phys. Rev. B* **76**, 153410, (2007).

2. Falkovsky, L. a. & Varlamov, a. a. Space-time dispersion of graphene conductivity. *Eur. Phys. J. B* **56**, 281-284, (2007).

3. Chen, J. *et al.* Optical nano-imaging of gate-tunable graphene plasmons. *Nature* **487**, 77-81, (2012).

4. Yeh, P. *Optical Waves in Layered Media*. (Wiley-Interscience, 2005).

5. Stratton, A. *Electromagnetic theory*. (Adams Press, 2008).

6. Cincotti, G. *et al.* Plane wave expansion of cylidrical functions. *Opt. Commun.* **95**, 192-198, (1993).

7. Borghi, R., Frezza, F., Santarsiero, M. & Schettini, G. Angular spectrum of modified cylindrical wave-functions. *Int. J. Infrared Millimeter Waves* **20**, 1795-1801, (1999).

8. Pfeiffer, C. a., Economou, E. N. & Ngai, K. L. Surface polaritons in a circularly cylindrical interface: Surface plasmons. *Phys. Rev. B* **10**, 3038-3051, (1974).

9. Wang, S. *et al.* Logarithm Diameter Scaling and Carrier Density Independence of One-Dimensional Luttinger Liquid Plasmon. *Nano Letters* **19**, 2360-2365, (2019).

10. Giuliani, G., Vignale, G. Quantum Theory of the Electron Liquid. Cambridge University Press: Cambridge, pp 501-549, (2005).

11. das Sarma, S. & Hwang, E. H. Dynamical response of a one-dimensional quantum-wire electron system. *Phys. Rev. B* **54,** 1936-1946, (1996).

12. Zhao, S. *et al*. Correlation of electron tunneling and plasmon propagation in a Luttinger liquid. *Physical Review Letters*, **121**, 047702, (2018).

13. Bockrath, M. *et al*. Luttinger-liquid behaviour in carbon nanotubes. *Nature* **397**, 598-601, (1999).

14. Kane, C., Balents, L., & Fisher, M. P.. Coulomb interactions and mesoscopic effects in carbon nanotubes. *Physical Review Letters*, **79**, 5086, (1997).

15. Woessner, A. *et al.* Propagating Plasmons in a Charge-Neutral Quantum Tunneling Transistor. *ACS Photonics* **4**, 3012-3017, (2017).
